# Supplementary material for: High Immunogenicity to Influenza Vaccination in Crohn’s Disease Patients Treated with Ustekinumab
Source: Vaccines (Basel). 2020 Aug 14;8(3):455. doi: 10.3390/vaccines8030455 (PMC7565576; doi:10.3390/vaccines8030455)
Supplement: Supplementary file 1 [file vaccines-08-00455-s001.zip › Figure S5. Correlation plots HI and SI.pdf]

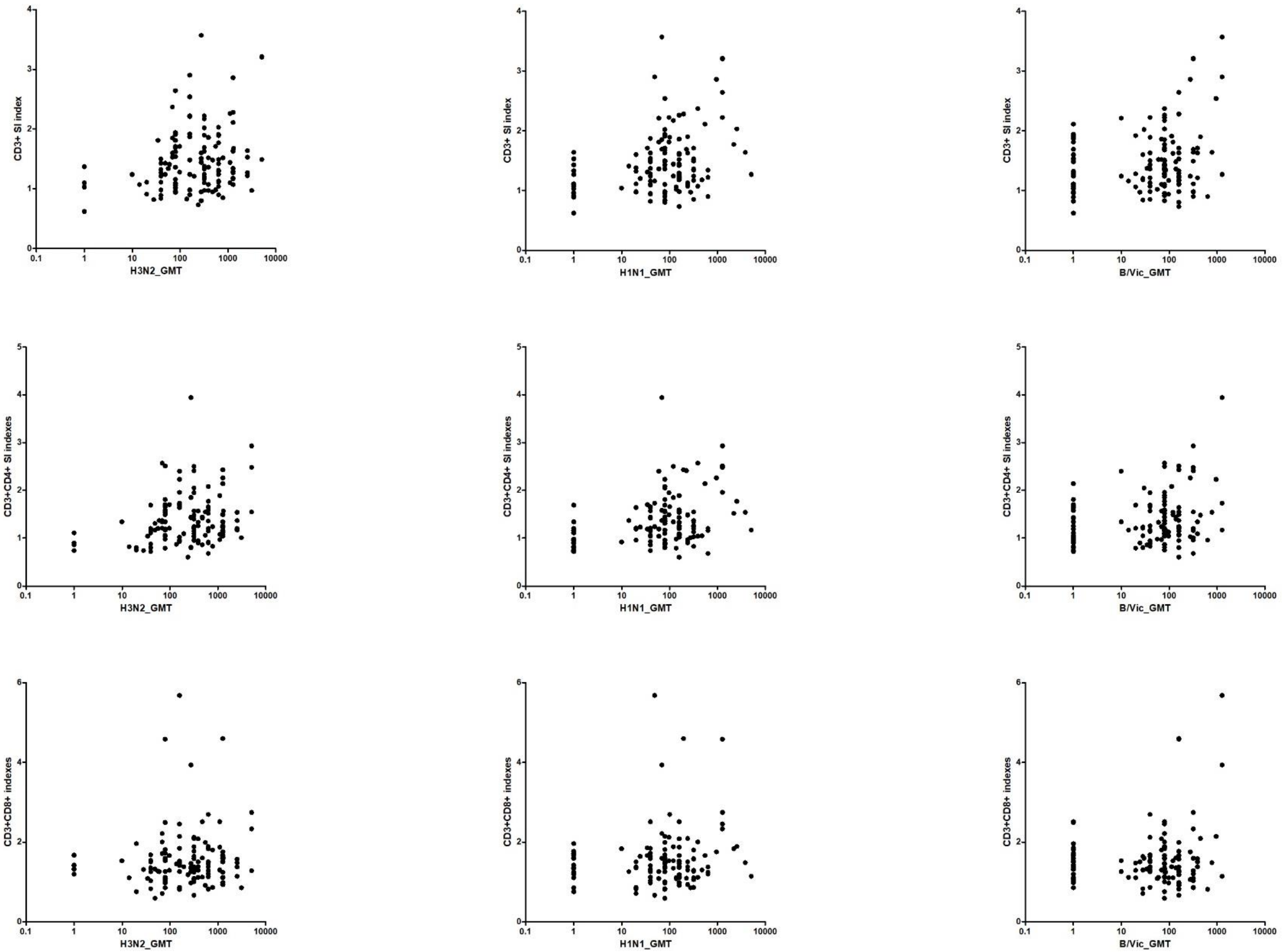

Supplementary figure 5: Scatter plots showing correlation between geometric mean titres and the stimulation indexes.
GMT = geometric mean titre, SI = stimulation index
