# Supplementary material for: High Immunogenicity to Influenza Vaccination in Crohn’s Disease Patients Treated with Ustekinumab
Source: Vaccines (Basel). 2020 Aug 14;8(3):455. doi: 10.3390/vaccines8030455 (PMC7565576; doi:10.3390/vaccines8030455)
Supplement: Supplementary file 1 [file vaccines-08-00455-s001.zip › Figure S4. Scatter plots of SI excluding outliers.pdf]

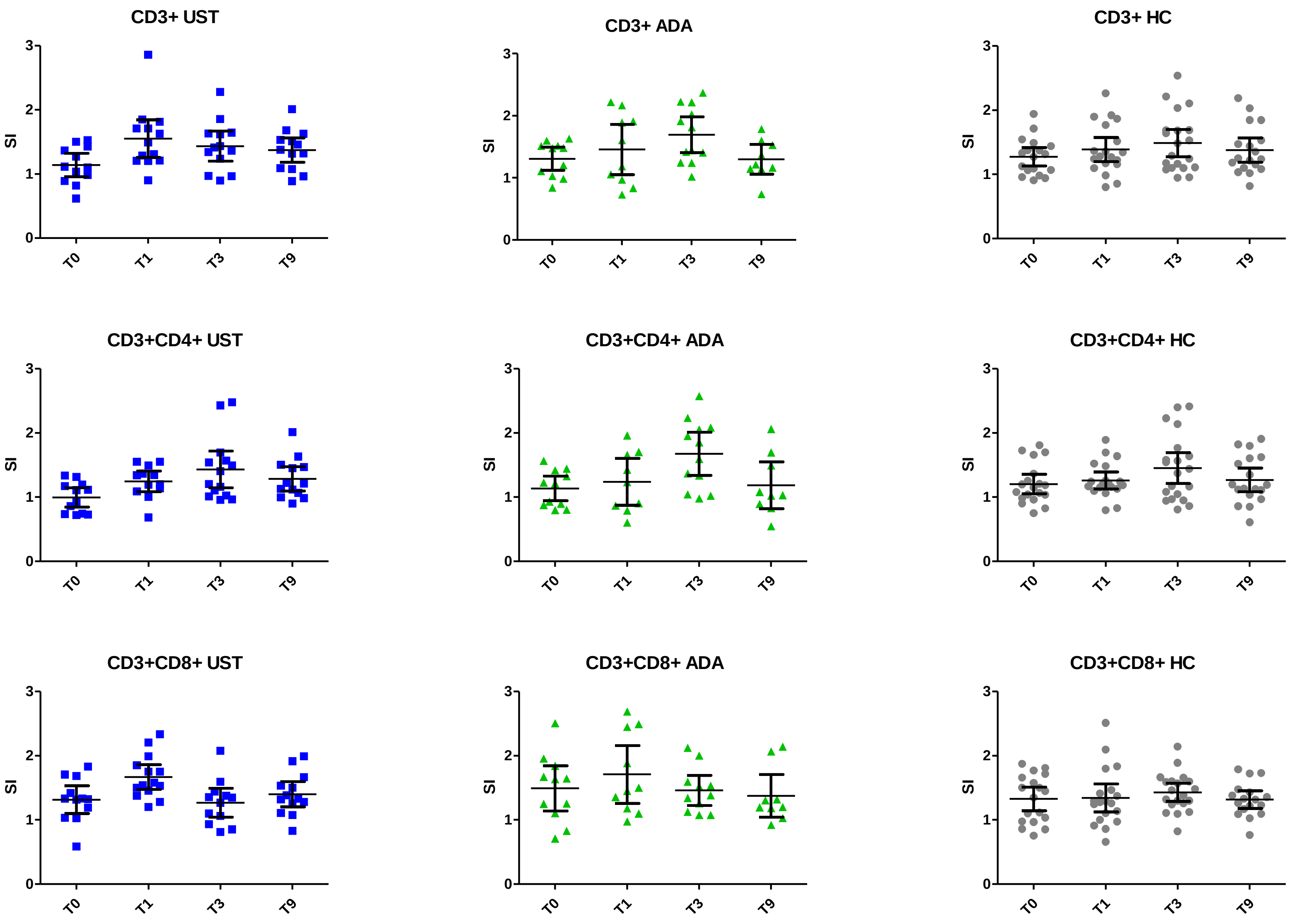

CD3+ UST
CD3+ ADA
CD3+ HC
CD3+CD4+ UST
CD3+CD4+ ADA
CD3+CD4+ HC
CD3+CD8+ UST
CD3+CD8+ ADA
CD3+CD8+ HC
SI
0
1
2
3
T0
T1
T3
T9

Suppl. Figure 4. Scatter plots of SI excluding outliers

SI = stimulation index, UST = ustekinumab users, ADA = adalimumab users, HC = healthy controls. 95% confidence intervals are shown. Outliers were detected with the Tukey's box-plot method which defines outliers as being outside the interquartile interval (Q1 – 1.5·IQR, Q3 + 1.5·IQR). The following outliers are excluded: COVA-207 (UST) T0 CD3/4/8, T1 CD4, T3 CD8, T9 CD3/4/8; COVA-208 (UST) T1 CD3/4, T3 CD3/8; COVA-209 (ADA) T3 CD8, T9 CD3/4/8, COVA-225 (HC) T0 CD3/CD8, T1 CD3/4/8, T9 CD8; COVA-239 (ADA) T1 CD4.
