# Supplementary figures and images for: High Immunogenicity to Influenza Vaccination in Crohn’s Disease Patients Treated with Ustekinumab
Source: Vaccines (Basel). 2020 Aug 14;8(3):455. doi: 10.3390/vaccines8030455 (PMC7565576; doi:10.3390/vaccines8030455)

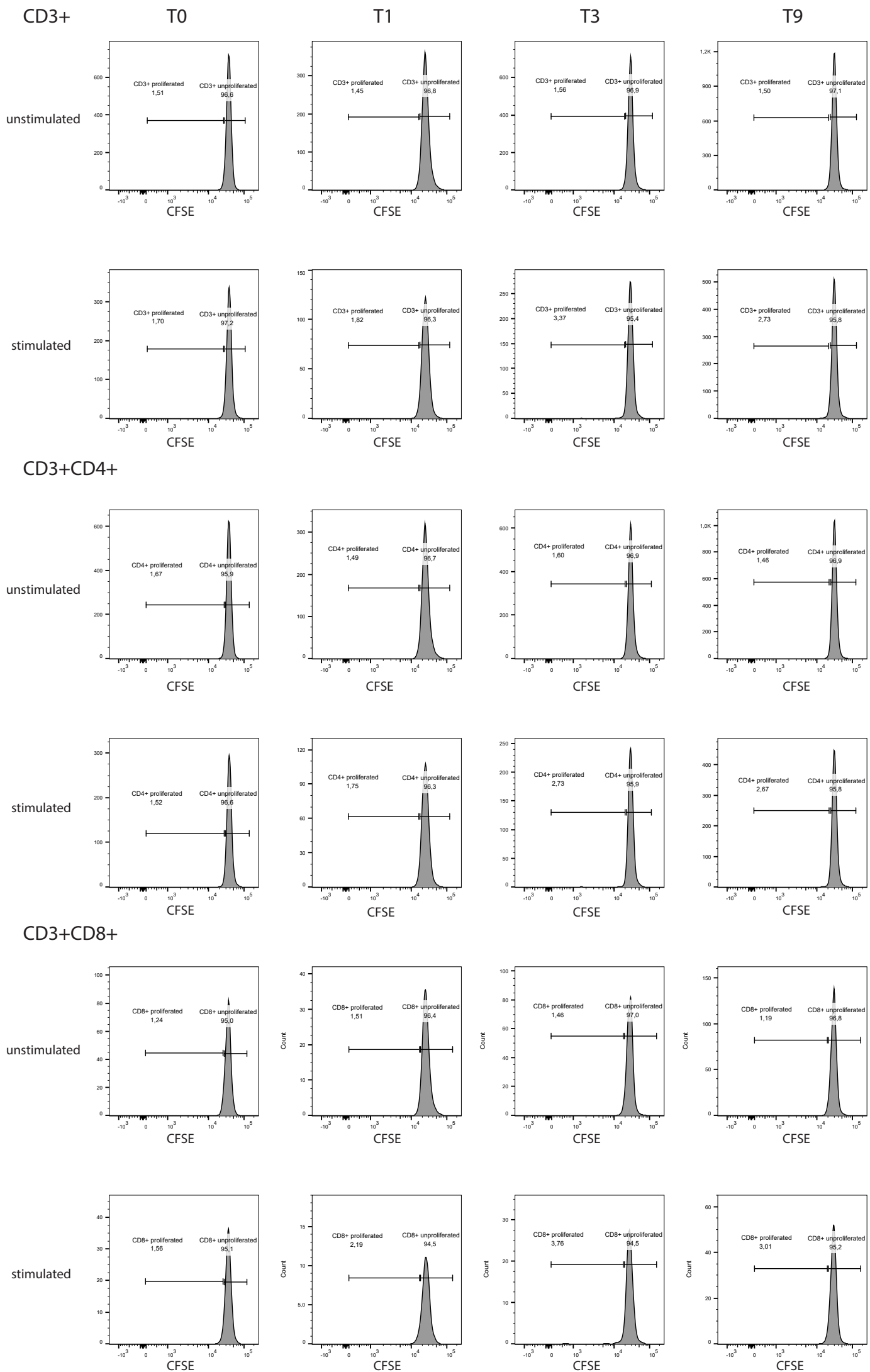

Supplement: Supplementary file 1 [file vaccines-08-00455-s001.zip › Figure S2. Example T-cell proliferation.pdf]

Supplementary Figure 1. Gating strategy on a sample stimulated with conA

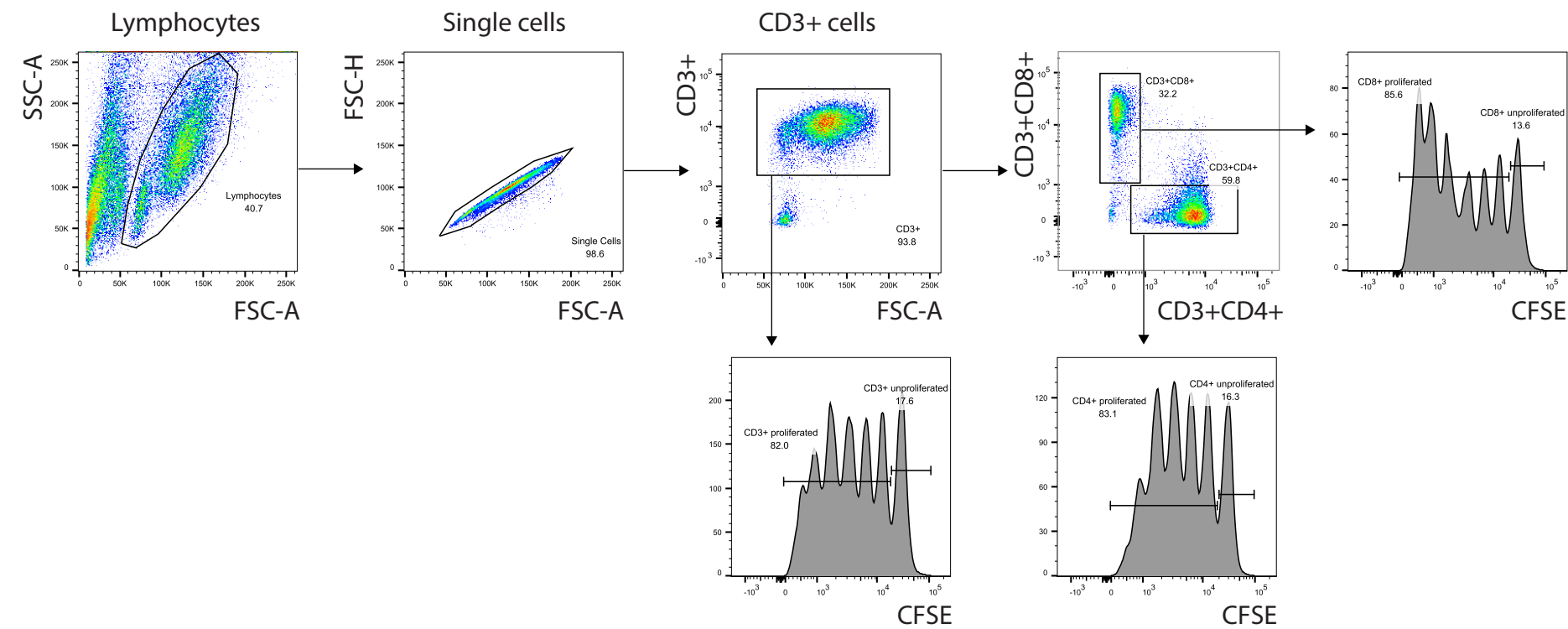

Supplement: Supplementary file 1 [file vaccines-08-00455-s001.zip › Figure S1. Gating strategy ConA.pdf]
